# Correction: Quantum-inspired pedestrian mobility modeling: Applying probabilistic spatial simulation to urban walkability and thermal comfort in Sri Lanka

**DOI:** 10.1371/journal.pone.0352177

**Published:** 2026-06-22

**Authors:** Malith Deshan, Amila Jayasinghe, Chethika Abenayake

The corresponding author’s email address is incorrect. Amila Jayasinghe’s email address is: amilabj@uom.lk.
