# Supplementary material for: Quantum-inspired pedestrian mobility modeling: Applying probabilistic spatial simulation to urban walkability and thermal comfort in Sri Lanka
Source: PLoS One. 2026 May 26;21(5):e0348630. doi: 10.1371/journal.pone.0348630 (PMC13210383; doi:10.1371/journal.pone.0348630)
Supplement: S1 Table — (DOCX) [file pone.0348630.s001.docx]

**Table S1.** Pearson correlation matrix of built-form, movement, and microclimatic covariates

Pearson correlation matrix for built-form, movement, and microclimatic covariates. Several indicators cluster into strongly collinear groups (e.g. building density–building height–shadow intensity; pedestrian/POI/vehicle densities; isovist–road accessibility–street centrality–wall constraints), motivating the proxy-based variable specification described in Section 4.1. Spearman correlations (not shown) exhibit the same grouping pattern.

| **Variable** | **Building**  **density** | **Building**  **height** | **shadow**  **intensity_mean** | **isovist** | **mean_depth** | **pedestrian_accessibility** | **pedestrian_density** | **poi_density** | **road_accessibility** | **street_centrality** | **tree_height** | **vehicle_density** | **wall_constraint** |
| --- | --- | --- | --- | --- | --- | --- | --- | --- | --- | --- | --- | --- | --- |
| **building_density** | 1.000 | 1.000 | 0.779 | -0.114 | -0.312 | -0.287 | 0.041 | 0.041 | -0.114 | -0.114 | -0.103 | 0.041 | -0.114 |
| **building_height** | 1.000 | 1.000 | 0.779 | -0.114 | -0.312 | -0.287 | 0.041 | 0.041 | -0.114 | -0.114 | -0.103 | 0.041 | -0.114 |
| **shadow_**  **intensity_mean** | 0.779 | 0.779 | 1.000 | -0.002 | -0.240 | -0.230 | 0.066 | 0.066 | -0.002 | -0.002 | 0.380 | 0.066 | -0.002 |
| **isovist** | -0.114 | -0.114 | -0.002 | 1.000 | 0.069 | 0.277 | 0.076 | 0.076 | 1.000 | 1.000 | 0.269 | 0.076 | 1.000 |
| **mean_depth** | -0.312 | -0.312 | -0.240 | 0.069 | 1.000 | 0.184 | 0.043 | 0.043 | 0.069 | 0.069 | 0.149 | 0.043 | 0.069 |
| **pedestrian_**  **accessibility** | -0.287 | -0.287 | -0.230 | 0.277 | 0.184 | 1.000 | 0.385 | 0.385 | 0.277 | 0.277 | 0.024 | 0.385 | 0.277 |
| **pedestrian_**  **density** | 0.041 | 0.041 | 0.066 | 0.076 | 0.043 | 0.385 | 1.000 | 1.000 | 0.076 | 0.076 | 0.050 | 1.000 | 0.076 |
| **poi_density** | 0.041 | 0.041 | 0.066 | 0.076 | 0.043 | 0.385 | 1.000 | 1.000 | 0.076 | 0.076 | 0.050 | 1.000 | 0.076 |
| **road_**  **accessibility** | -0.114 | -0.114 | -0.002 | 1.000 | 0.069 | 0.277 | 0.076 | 0.076 | 1.000 | 1.000 | 0.269 | 0.076 | 1.000 |
| **street_centrality** | -0.114 | -0.114 | -0.002 | 1.000 | 0.069 | 0.277 | 0.076 | 0.076 | 1.000 | 1.000 | 0.269 | 0.076 | 1.000 |
| **tree_height** | -0.103 | -0.103 | 0.380 | 0.269 | 0.149 | 0.024 | 0.050 | 0.050 | 0.269 | 0.269 | 1.000 | 0.050 | 0.269 |
| **vehicle_density** | 0.041 | 0.041 | 0.066 | 0.076 | 0.043 | 0.385 | 1.000 | 1.000 | 0.076 | 0.076 | 0.050 | 1.000 | 0.076 |
| **wall_constraint** | -0.114 | -0.114 | -0.002 | 1.000 | 0.069 | 0.277 | 0.076 | 0.076 | 1.000 | 1.000 | 0.269 | 0.076 | 1.000 |

Note: Several indicators cluster into strongly collinear groups (e.g. building density–building height–shadow intensity; pedestrian/POI/vehicle densities; isovist–road accessibility–street centrality–wall constraints), motivating the proxy-based variable specification described in Section 4.1. Spearman correlations (not shown) exhibit the same grouping pattern
