# Supplementary material for: Quantum-inspired pedestrian mobility modeling: Applying probabilistic spatial simulation to urban walkability and thermal comfort in Sri Lanka
Source: PLoS One. 2026 May 26;21(5):e0348630. doi: 10.1371/journal.pone.0348630 (PMC13210383; doi:10.1371/journal.pone.0348630)
Supplement: S2 Table — (DOCX) [file pone.0348630.s002.docx]

**Table S2.** Variance inflation factors (VIFs) for spatial covariates

.

Variance inflation factors (VIFs) for spatial covariates. Several variables display numerically infinite VIFs in the software output, indicating near-perfect multicollinearity and confirming that many indicators are structurally coupled (e.g. density-related and network-based measures). Variables with moderate VIF values (shadow intensity, tree height, pedestrian accessibility, mean depth) were retained as primary proxies for microclimate, vegetation, accessibility, and configurational depth in the proxy-based specification.

| **Variable** | **VIF value** |
| --- | --- |
| building_density | ∞ |
| building_height | ∞ |
| isovist | ∞ |
| pedestrian_density | ∞ |
| poi_density | ∞ |
| road_accessibility | ∞ |
| street_centrality | ∞ |
| vehicle_density | ∞ |
| wall_constraint | ∞ |
| shadow_intensity_mean | 5.694 |
| tree_height | 2.433 |
| pedestrian_accessibility | 1.428 |
| mean_depth | 1.158 |

Note: Several variables display numerically infinite VIFs in the software output, indicating near-perfect multicollinearity and confirming that many indicators are structurally coupled (e.g. density-related and network-based measures). Variables with moderate VIF values (shadow intensity, tree height, pedestrian accessibility, mean depth) were retained as primary proxies for microclimate, vegetation, accessibility, and configurational depth in the proxy-based specification.
