# Supplementary material for: Quantum-inspired pedestrian mobility modeling: Applying probabilistic spatial simulation to urban walkability and thermal comfort in Sri Lanka
Source: PLoS One. 2026 May 26;21(5):e0348630. doi: 10.1371/journal.pone.0348630 (PMC13210383; doi:10.1371/journal.pone.0348630)
Supplement: S3 Table — (DOCX) [file pone.0348630.s003.docx]

**Table S3.** Five-fold spatial stability analysis for the quantum-inspired model.

| **Fold** | **n** | **Pearson r** | **Spearman ρ** | **RMSE** |
| --- | --- | --- | --- | --- |
| 1 | 340 | 0.676 | 0.6483 | 32,450 |
| 2 | 342 | 0.6596 | 0.612 | 30,100 |
| 3 | 342 | 0.7092 | 0.681 | 37,800 |
| 4 | 342 | 0.6349 | 0.6955 | 36,700 |
| 5 | 339 | 0.6168 | 0.5668 | 35,800 |

**Summary across folds**

| **Metric** | **Mean** | **SD** |
| --- | --- | --- |
| Pearson r | 0.659 | 0.036 |
| Spearman ρ | 0.638 | 0.054 |
| RMSE | 34,556 | 3,266 |
